# A model-based approach to automation of formal verification of ROS 2-based systems

**DOI:** 10.3389/frobt.2025.1592523

**Published:** 2025-07-16

**Authors:** Lukas Dust, Rong Gu, Saad Mubeen, Mikael Ekström, Cristina Seceleanu

**Affiliations:** School of Innovation, Design, and Technology, Mälardalen University, Västerås, Sweden

**Keywords:** ROS 2, robotic systems, formal verification, model checking, model-based engineering

## Abstract

Formal verification of robotic applications, particularly those based on ROS 2, is desirable for ensuring correctness and safety. However, the complexity of formal methods and the manual effort required for model creation and parameter extraction often hinder their adoption. This paper addresses these challenges by proposing a model-based methodology that automates the formal verification process using model-driven engineering techniques. We introduce a methodology which can be applied as a toolchain that automates the initialization of formal model templates in UPPAAL using system parameters derived from ROS 2 execution traces generated by the ROS2_tracing tool. The toolchain employs four model representations based on custom Eclipse Ecore metamodels to capture both structural and verification aspects of ROS 2 systems. The methodology supports both implemented and conceptual systems and enables iterative verification of timing and scheduling parameters through model-to-model and model-to-text transformations. A proof-of-concept implementation demonstrates the feasibility of the proposed approach. The designed toolchain supports verification using two types of UPPAAL models: one for individual node verification (e.g., callback latency and buffer overflow) and another for end-to-end latency analysis of ROS 2 processing chains. Experiments conducted on two implemented and one conceptual ROS 2 systems validate the correctness and adaptability of the toolchain. The results show that the toolchain can automate parameter extraction and model generation. The proposed methodology modularizes the verification process, allowing domain experts to focus on their areas of expertise. It targets to enhances traceability and reusability across different verification scenarios and formal models. The approach aims to make formal verification more accessible and practical to robotics developers.

## 1 Introduction

Ensuring that a robotic system’s design and implementation meet the requirements specification is crucial for guaranteeing the system’s desired behavior. Various verification methods are employed to achieve this, with formal methods, e.g., model checking, being particularly effective due to their rigorous mathematical approach to analyzing complex system models during the design phase (Carvalho et al., 2020). Model checking involves an exhaustive exploration of the system’s model state space to verify that the system meets its specification, uncovering potential errors that might be missed by traditional trial-and-error methods such as simulation and experimentation (Dust et al., 2023a).

Despite its advantages, the application of formal model-based approaches in distributed and complex systems poses significant challenges. The steep learning curve associated with the mathematical syntax and semantics of formal modeling languages can be a barrier for robotic developers. Consequently, the high initial effort required for model checking often deters its use in industry, leading developers to rely on less rigorous, trial-and-error methods (Rajkumar et al., 2010).

The Robot Operating System (ROS) (OpenRobotics, 2023b; OpenRobotics, 2023a) is an open-source middleware that facilitates rapid prototyping and deployment of robotic systems. ROS-based systems, particularly those with safety-critical applications, have stringent timing requirements that necessitate real-time capabilities in the middleware. These capabilities are influenced by various system components, including communication, task scheduling, and execution. To address the limitations of ROS in real-time applications, ROS 2 was developed, incorporating real-time communication through the Data Distribution Service (DDS) C. S. V. (Gutiéerrez et al., 2018). While DDS provides a robust framework for real-time communication, the task scheduling in ROS 2 still requires extensive analysis to ensure deterministic timing behavior (Casini et al., 2019; Blaß et al., 2021).

Tools like *ROS2_tracing* (Bédard et al., 2022) and *Autoware_perf* (Li et al., 2022) have been developed to trace system execution and analyze performance based on execution traces. However, these tools primarily offer experimental analysis, which may not be exhaustive and could miss potential system errors. In contrast, model checking offers a comprehensive verification approach capable of identifying all potential bugs in the model. Despite this, the manual application of formal methods to ROS 2 systems remains error-prone and time-consuming, requiring significant background knowledge.

In our previous work (Dust et al., 2023a), we utilize the UPPAAL model checker (Alur and Dill, 1994) to create reusable templates for verifying timing behavior and buffer overflow in ROS 2 systems. These templates simplify the modeling process by allowing systems to be instantiated from pre-defined templates rather than constructed from scratch. However, this approach still requires detailed knowledge of static and runtime system parameters, as well as of the modeling language itself, to represent verification properties accurately. The manual nature of this process makes it susceptible to errors, as parameters are often determined through source-code analysis and runtime evaluation.

### 1.1 Problem definition and paper contributions

In our previous work (Dust et al., 2023b; Dust et al., 2024), we identified scheduling-related timing issues in ROS 2 and developed formal model templates to address such issues (Dust et al., 2023a). The proposed template-based verification facilitates formal verification, but initializing the formal model templates requires extended knowledge and analysis of static and runtime parameters. Furthermore, due to the manual process of initializing the formal models, extended knowledge about the modeling language is needed. Additionally, manual source code and runtime analysis make the proposed verification vulnerable to errors. In this paper, we aim to simplify the formal verification process by proposing a model-based methodology that automates parameter determination and model initialization using the existing formal model templates. This methodology is designed for robotics developers, with the goal of making formal verification more accessible and less error-prone. In this article, we extend our work (Dust et al., 2024), automating model-based formal verification using model-driven engineering techniques.

Based on the problem definition, we develop the three research questions. The research questions are presented in the following paragraphs.

As the first goal of this paper, we aim to identify an approach that can be used to automate the application of formal verification. By proposing an approach, we aim to reduce the complexity for practitioners when applying formal methods through the facilitation of automation. To achieve the stated goal, we design a methodology and implement a proof of concept of a toolchain that enables the application of such methodology. Hence, we contribute a bridging approach utilizing ROS 2 traces, modeling, and formal methods that automate formal verification through automated transformations.

As the second goal of this paper, we aim to modularize the process of formal verification. The modularization targets to decouple components of the verification process to enable actors to focus on their domain of expertise in the creation and adaptation of the verification process. In this paper, we modularize the process of formal verification through the design and modeling of a methodology that we apply in a novel toolchain implemented in this paper. Hence, we propose a layered and modular methodology that can be applied through the implemented toolchain.

As the third goal of the paper, we aim to propose a methodology that allows verification of different formal models, focusing on different properties to verify. The proposed methodology aims to separate the concerns in terms of the type of properties to verify. We demonstrate the ability of the methodology to allow verification of different formal models by implementation of a toolchain and experimental evaluation. As a result of the design, implementation, and evaluation, we develop a novel validated UPPAAL model for end-to-end (E2E) latency to enable comparison to formal models proposed in the literature. Furthermore, we develop a proof of concept that covers multiple verification goal-oriented UPPAAL models.

Summarizing, the following research questions are tackled in this paper:

**RQ1:** What approach can be employed to automate the application of formal verification of ROS 2-based applications?
**RQ2:** How can the formal verification process be modularized to enable domain experts to concentrate on their specific areas of expertise without requiring deep formal methods knowledge?
**RQ3:** How can a methodology incorporate verification using different formal models?

The contributions of this paper are summarized as follows:

1. A novel methodology for model-based verification of ROS 2 systems, featuring a toolchain that includes UPPAAL, Eclipse, and *ROS2_tracing.*
2. UPPAAL models for verification of end-to-end latencies in ROS 2 processing chains in a single executor.
3. Ecore metamodels to capture system structure and support verification activities.
4. Automated and conceptual model-to-model transformations from ROS 2 execution traces to Ecore models, and from Ecore models to UPPAAL models.
5. Demonstration of the proposed toolchain’s workflow through a proof of concept implementation, covering key aspects of the toolchain architecture.

The remainder of this paper is organized as follows. Section 2 provides an overview of model checking, ROS 2, and model-driven engineering using Eclipse. Section 3 details the proposed methodology and toolchain architecture, along with the potential workflow. Section 4 presents the proof of concept implementation, followed by a discussion of related work in Section 6. The paper concludes with final remarks, and prospective future work in Section 7.

## 2 Background

In this section, we provide an overview of the essential concepts and tools relevant to our work, including model checking, ROS 2, model-driven engineering using Eclipse, and end-to-end timing analysis.

### 2.1 Model checking and UPPAAL

*Model checking* is a formal verification technique that offers a rigorous, mathematical approach to the analysis of complex systems during the design phase (Carvalho et al., 2020). It involves an exhaustive exploration of the system’s model state space to ensure that the system meets its specification. UPPAAL is a widely used model checker for the modeling, simulation, and verification of real-time systems described as *timed automata* (Alur and Dill, 1994). It supports the creation of reusable templates to verify timing behavior and buffer overflow, making the application of formal verification more accessible.

Below, we provide a brief, informal overview of timed automata (TA). For detailed and precise definitions of TA and their application in UPPAAL, we refer the reader to the literature (Alur and Dill, 1994; Hendriks et al., 2006).

A *timed automaton* (TA) (Alur and Dill, 1994) consists of a finite set of *locations*, including an initial location, which are connected by *edges*, as well as a finite set of non-negative real-valued variables, known as *clocks*, which measure the elapse of time and progress simultaneously at rate 1. The edges are decorated with a finite set of *actions*, and *guards*, which are conjunctive Boolean formulas of clock constraints that need to evaluate to *true* for the edge to be traversed. Clocks can be reset over the edges, and a partial function assigns *invariants* to locations, which constrain the time allowed to elapse in a particular location. The semantics of TA is defined as a *labeled transition system* with delay and action transitions.

**FIGURE 1:**
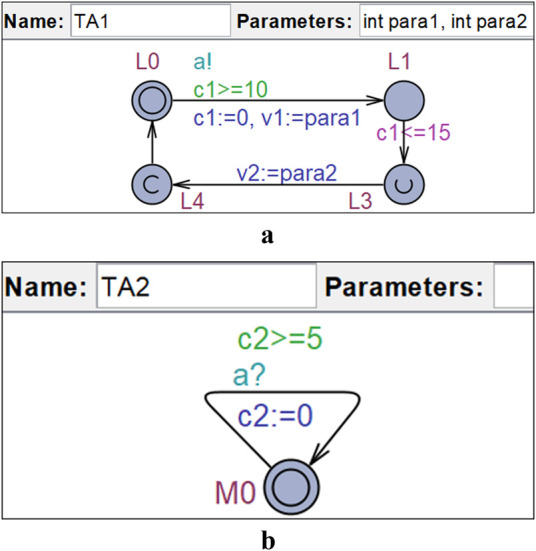
Examples of UTA in UPPAAL. **(a)** UTA template *TA1.* **(b)** UTA template *TA2.*

UPPAAL (Hendriks et al., 2006) is a tool used for modeling, simulation, and model checking of an extended version of timed automata called *UPPAAL Timed Automata* (UTA). In UPPAAL, UTA are organized as *templates* (see Figure 1) that can be instantiated. UTA enhances the capabilities of TA by adding features such as data variables, synchronization channels (Boolean variables decorated by “!” for sending, and by “?” for receiving), urgent and committed locations, and more. Furthermore, UPPAAL allows the composition of UTA in parallel as a network of UTA (NUTA), synchronized via *channels*.

Figures 1a,b show two NUTA implemented in UPPAAL. In these figures, blue circles represent *locations* connected by directional *edges*. Double-circled locations are the *initial* locations (e.g., L0). Locations marked with an encircled ”u” are *urgent* (e.g., L3), and those with an encircled ”c” are *committed* (e.g., L4). UTA imposes constraints that prevent time from progressing in urgent and committed locations. Committed locations have stricter rules: the next edge traversal must start from one of them.

Edges allow for assignments such as resetting clocks (e.g., c1≔0), updating data variables (e.g., v1≔para1), guards (e.g., c1>=10), and synchronization channels (e.g., a! and a?). At location L1, an invariant c1<=15 ensures that clock c1 does not exceed 15 time units in that location. In UPPAAL, UTA templates can include parameters (e.g., para1 in TA1) that are assigned values upon instantiation.

### 2.2 ROS 2

The Robot Operating System 2 (ROS 2) (OpenRobotics, 2023a) is an open-source middleware designed to facilitate the rapid development and prototyping of robotic systems. Unlike what its name suggests, ROS 2 is not a standalone operating system but rather runs on top of an existing host OS, predominantly Linux.

ROS 2 was developed to meet industrial requirements such as fault tolerance and real-time performance. To achieve real-time capabilities, ROS 2 introduced the Data Distribution Service (DDS) Group (2022) as its communication protocol. DDS, created by the Object Management Group (OMG) Group (2022), enables efficient communication between distributed applications. While DDS is the default communication protocol, other protocols such as Zenoh can be utilized.

The fundamental building blocks of ROS 2 systems are *nodes*, which communicate through designated channels using DDS. ROS 2 supports two primary communication paradigms (Birman and Joseph, 1987): *Publisher-Subscriber* and *Service-Client*. In the *Publisher-Subscriber* model, nodes can either *publish* messages to a specific topic or *subscribe* to receive messages from that topic. All nodes subscribed to a topic receive the published messages. Conversely, the *Service-Client* model involves directed communication, where a client node requests a service from a server node, which then processes the request and sends back a response.

**FIGURE 2:**
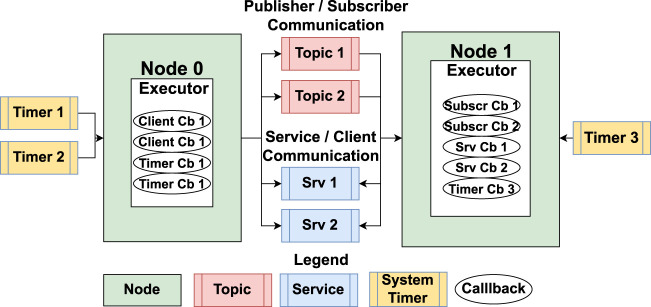
An example ROS 2 system showing the concepts of Node, Timer, Topic, Publisher, Subscriber, Service, and Client.

Figure 2 illustrates an example of a ROS 2 system with two nodes communicating over four channels. In addition to communication channels, system timers can be used to trigger functions within a node at specified intervals.

Nodes in ROS 2 are executable entities within the host OS and consist of several functions known as *callbacks*, which are the atomic schedulable units in ROS 2. Callbacks are triggered by events such as the arrival of data in an input buffer or a timer event. There are four types of callbacks: timer, subscriber, service, and client.

Each node also includes an executor, which is responsible for scheduling and executing callbacks (Casini et al., 2019; Blaß et al., 2021). The executor can operate with single or multiple threads, depending on the configuration chosen. However, the latest versions of ROS 2 do not provide options to set callback priorities, making the execution susceptible to blocking. This can lead to issues such as buffer overflow and missed callback instances in worst-case scenarios (Dust et al., 2023b; Dust et al., 2023a).

### 2.3 ROS2_tracing

ROS2_tracing (Bédard et al., 2022) is a low-overhead framework based on the Linux Trace Toolkit next-generation (LTTng). It is included in the ROS 2 installation and allows for the generation of execution traces. These traces provide valuable insights into the system’s behavior, including callback execution times, message passing instances, and system initialization events. The ROS2_tracing toolbox includes a Python library called tracetools_analysis, which transforms LTTng traces into a defined ROS 2 data model, represented as pandas Python objects (Bédard et al., 2022).

### 2.4 Autoware real Time reference system

The Autoware Reference System (ROS Realtime Working Group, 2025b) is part of the ROS 2 (Robot Operating System) ecosystem, specifically designed to provide a standardized and repeatable benchmarking environment for evaluating the performance of various executors and configurations within the ROS 2 framework (ROS Realtime Working Group, 2025a). This real-time reference system simulates the Autoware. Auto (Autoware Foundation, 2025) LiDAR data pipeline, to measure and compare the performance of different executor implementations. The reference system is defined by a fixed number of nodes, each with specific publishers, subscribers, processing times, and publishing rates. It uses a fixed message type and size for consistency. The system can run on various platforms, including different hardware and operating systems, ensuring that the benchmarks are portable and replicable.

### 2.5 Pattern-based verification of ROS 2 timing

In our previous work (Dust et al., 2023a), we proposed a pattern-based verification approach for analyzing the execution behavior of ROS 2 systems using UPPAAL. This approach focuses on verifying two key properties: callback latency and input buffer sizes.

**Callback Latency**: This is defined as the maximum time between the release of a callback instance and the completion of its execution. Ensuring low callback latency is crucial for maintaining the responsiveness of the system.

**Input Buffer Size**: This property verifies that the input buffer is large enough to handle incoming data for a given system configuration. Adequate buffer sizes prevent data loss and ensure smooth data flow within the system.

To facilitate the verification process, we create three types of UPPAAL templates to represent ROS 2 nodes:

• **Wall-Time-Callbacks**: These are callbacks that are released at specific times.

• **Periodic Callbacks**: These are callbacks that are released periodically.

• **Executors**: These represent different versions of the ROS 2 executor, which schedules and executes callbacks.

These templates can be composed to model a ROS 2 system, allowing for exhaustive verification of timing properties. Listing 1 illustrates an example of UPPAAL system initialization:

Listing 1 Example of UPPAAL System Initialization.

// Executor start_exp
// Release Times for WallTimeCallbacks
const int releasesSUBSCRIBER0[MAXX]={43,43,43,43,0,0,0,0,0,0};
// Executor
ExV1 = ExecutorExV1(StopTime);
// Callbacks
SUBSCRIBER0 = WallTimeCallback(0,5,4,releasesSUBSCRIBER0,SUBSCRIBER,1000);
// System Definition
system ExV1 < SUBSCRIBER0;

The listing above shows an example of how a ROS 2 system can be initialized using UPPAAL templates. In this example:

• An array is created to hold the release times for a Wall-Time-Callback. In this case, the callback is released four times at 43 m intervals.

• An executor is initialized with a defined stop time, which describes the interval for which the verification will be conducted.

• The Wall-Time-Callback template is instantiated with parameters such as the callback ID, execution time, number of releases, release time array, callback type, and buffer size.

• The system is defined as a composition of the executor and the callback.

This initialization process allows for the simulation and verification of the system’s timing behavior using UPPAAL. By modeling the system in this way, we can conduct exhaustive verification to ensure that the system meets its timing requirements and identify any potential issues related to callback latency and buffer sizes. The actual verification of such properties happens in the UPPAAL verifier through checking the states of defined variables.

The pattern-based verification approach provides a structured and systematic method for analyzing the timing behavior of ROS 2 systems, making it easier for developers to ensure the correctness and reliability of their systems.

### 2.6 Eclipse modeling framework (EMF)

The Eclipse Modeling Framework (EMF) (Steinberg et al., 2008) is a widely used tool for model-driven engineering (MDE). EMF provides a framework for defining metamodels and generating code from models. It supports model-to-model and model-to-text transformations, enabling the automation of various development tasks. In our work, we utilize EMF to create metamodels that represent different abstractions of ROS 2 systems, facilitating the automation of formal verification. EMF allows the definition of metamodels that are instances of the Ecore metamodel, which can be used to create models representing system components and their interactions.

### 2.7 End-to-end timing analysis

End-to-end timing analysis is crucial for ensuring the correct functionality and safety of autonomous systems, particularly in real-time applications. It involves analyzing the timing behavior of cause-effect chains, which represent sequences of reactions from a cause (e.g., sensing) to an effect (e.g., actuation). Two key metrics in end-to-end timing analysis are the *maximum reaction time* (the maximum time for the system to react to an external input) and the *maximum data age* (the maximum time between sampling and the output being based on that sample) (Teper et al., 2022).

In ROS 2 systems, end-to-end timing analysis can be challenging due to the combination of time-triggered and event-triggered components. Existing methods for periodic and sporadic task systems are not directly applicable to ROS 2. Therefore, we propose new UPPAAL templates that resemble the end-to-end timing analysis presented in (Teper et al., 2022). These templates are used to model and verify the timing behavior of ROS 2 systems, to ensure that they meet the required timing constraints.

## 3 Proposed methodology

In this section, we present a methodology designed to automate the formal verification of ROS 2-based systems. The methodology integrates execution traces generated at runtime, model-driven development, and the composition of formal model declarations and verification. This methodology aims to decouple the process of formal modeling from system development while ensuring traceability, enabling users to apply formal verification without requiring extensive domain expertise.

### 3.1 Definition of users

The primary goal of this methodology is to simplify the verification process for robotic systems. The intended end users are robotics developers who may have limited knowledge of modeling and formal verification. Hence, the aim is to allow robotics engineers to perform formal verification with limited learning effort.

Practically, the methodology is designed to enable the design of an extensible toolchain, where domain experts, such as formal verification specialists and modeling engineers focus on the aspects where they can contribute most. Once such a toolchain following the developed methodology is developed, implemented, and set up, robotics engineers should be able to operate it with limited learning effort. The definition of users and maintainers of the toolchain is essential to analyze potential modularization, as stated in RQ2.

### 3.2 Architectural overview

The toolchain comprises four main architectural components.

1. System Implementation Layer
2. Tracing Layer
3. Modeling Layer
4. Verification Layer

**FIGURE 3:**
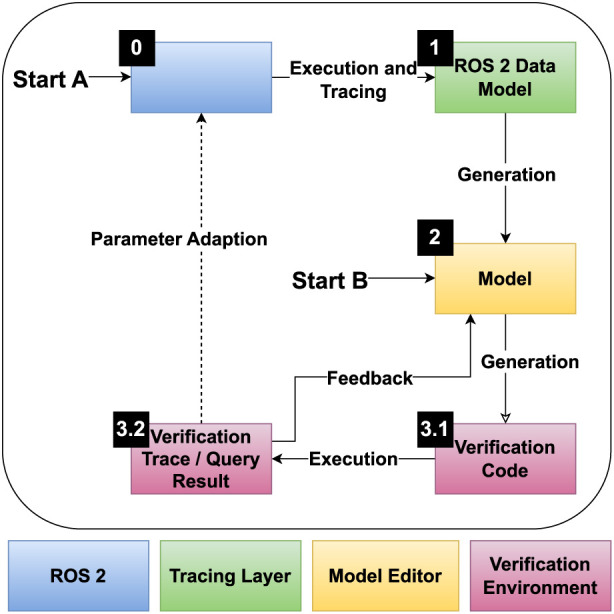
Architectural overview of the proposed methodology for automating verification of ROS 2-based systems. The architecture includes layers for system implementation, tracing, modeling, and verification.

**FIGURE 4:**
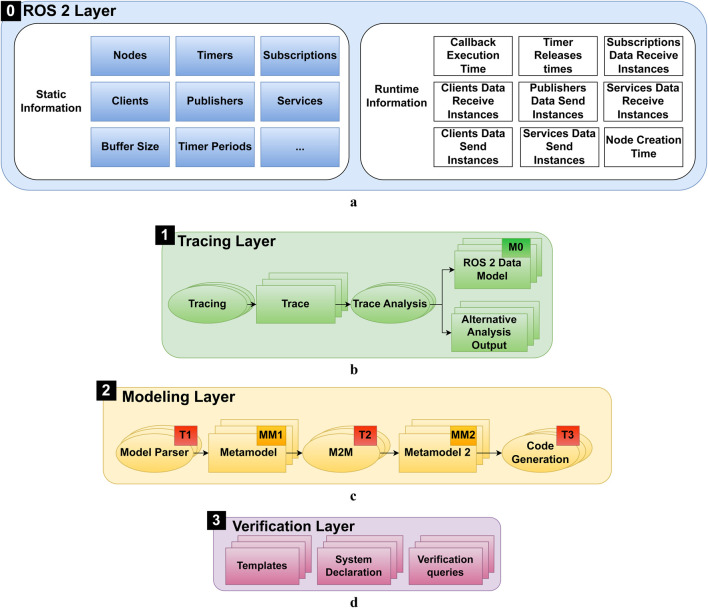
Detailed overview of the elements in the component layers. (1) System Implementation Layer. (2) Tracing Layer. (3) EMF Modeling Layer. (4) Verification Layer. **(a)** Overview of the ROS 2 system implementation, highlighting the static and dynamic information captured during system execution. **(b)** Tracing layer overview, showing the process of generating and analyzing execution traces to create the ROS 2 Data Model. **(c)** Modeling Layer overview, illustrating the different metamodels and transformations used to automate the verification process. M2M stands for model-to-model transformation. **(d)** Verification Layer overview, showing the generation of UPPAAL artifacts from EMF models for formal verification.

Figure 3 provides an overview of the methodology, showing the included layers and their connections. Each layer is marked in a different color. It can be seen that with Start A and Start B, there are two starting points for the potential application of the methodology. They refer to the two possible application approaches, namely, the verification of already implemented, executable systems, and the verification of conceptual system design. A more detailed overview of the components of each layer can be found in Figure 4. In the figures, MM stands for Meta-Model, M stands for Model, and T stands for Transformation. In Figure 4, boxes stand for artifacts and system components such as models and traces, while ellipses stand for actions such as transformations. The subsequent sections explain the layers in more detail.

#### 3.2.1 System Implementation Layer

The ROS 2 Layer 0 (light blue) represents the ROS 2 system implementation, encompassing both static information (Figure 4a) (dark blue) and dynamic information (white). Static information includes system components such as nodes, timers, subscriptions, publishers, services, and clients. Dynamic information involves runtime data such as callback execution times, timer release times, and message passing instances that describe the system behavior during execution.

#### 3.2.2 Tracing layer

The Tracing Layer 1 (green) utilizes tracing to generate execution traces from the running system. An overview of the elements in this layer is presented in Figure 4b. Generated traces contain both static and dynamic information, and are then transformed into a human-readable ROS 2 Data Model representation (M0). Additionally, customized analysis can be performed during the initial analysis of the traces, such as message flow analysis, as proposed in (Bédard et al., 2023). Based on the generated traces and analysis, a detailed visualization of system components and their interactions is possible. The traces are an important part to answer RQ1, as they enable automated determination of system parameters.

#### 3.2.3 Modeling layer

The Modeling Layer 2 (light yellow) shown in Figure 4c involves the use of a modeling framework to create and utilize different metamodels and transformations. The metamodels allow the definition of models to model the system from different perspectives. The following exemplary types of metamodels allow representation of various abstractions of the ROS 2 system:

• **MM1 Metamodel**: Input Metamodel: Maps the ROS 2 Data Model to an model for use in a model editor, allowing detailed analysis and traceability.

• **MM2 Metamodel 2**: System Model: Allows modeling of the system from a perspective towards the formal model. Parameters not needed for verification might be excluded, and component links might be made using a different approach. This model is used to decouple the formal modeling further from the tracing and parsing. Hence, if a different formal model is introduced, MM2 has to be exchanged. Nevertheless, there can be multiple different implementations of MM2 to allow the generation of different formal model representations.

While it is possible to generate the verification code from the traces directly, it is beneficial to include different metamodels during the generation steps. This allows traceability throughout the generation process, which might lead to a better understanding of parameters. Furthermore, this allows for the testing of different parameters without the need to adapt and rerun the system. Additionally, the introduction of incremental steps is essential to achieve modularization as stated in RQ2. Note that in a modeling environment, there might be multiple different implementations of MM2 to allow the application of different formal models.

To simplify the transformation between the different model representations, three types of transformations can be employed:

• **Model Parsing (T1)**: Transforms textual model descriptions into model representations specific to the chosen modeling environment, ensuring compatibility with the metamodel definitions.

• **Model to Model Transformation (T2)**: Converts one model representation to another, facilitating different sets of features and abstractions.

• **Code Generation (T3)**: Translates models into executable code, enabling the generation of formal model declarations and verification queries.

The shown transformations can be automated using designated tools. Such automation potential is important to answer RQ1.

#### 3.2.4 Verification layer

The Verification Layer 3 (violet) shown in Figure 4d contains three main elements. First of all, there are the formal model templates, which are created by a verification expert; the templates capture the behavior of the system components, formally. The templates can be composed into a system, in the system declaration. Using the toolchain, the system declaration can be produced through model-to-text transformations. Verification queries can be executed in the verifier. The verifier allows verification of the properties of a system specified by such queries. They can be predefined as templates and adapted to the chosen notation and naming during the model-to-text transformations. When applying the methodology following the architectural overview in Figure 3, upon creation of the formal model and queries in form of a compatible file (3.1), the outcome of the verification is given as potential execution traces and the query results (3.2). In the proposed methodology, multiple different implementations of Templates, Systems Declarations, and Verification Queries can be employed. This is essential to answer RQ3.

#### 3.2.5 Relation of components and modularization

In the previous subsections, we divided the methodology into four layers. Each of the layers consists of multiple components.

While the metamodels proposed in the Tracing Layer and the Modeling Layer are not strictly needed to enable automation, in this paper, they are introduced to enable modularization and decoupling of domain knowledge in the verification processes.

Generally, the boxes shown in Figures 4b–d show artifacts that result from specific actions shown as ellipses.

Two consecutive artifacts in the methodology are related by the fact that the set of attributes of the first artifact is an extension of the attributes of the second artifact. Hence, a transformation reduces the set of parameters while changing the structure of the model. As an example, one can transform an instance of the class Trace to an instance of the class ROS 2 Data Model by using Trace_analysis library functions. The classes are substitutable by any other class that obeys the extends mechanism. However, the transformation needs to be adapted, provided that the inherited attributes of the substituted class change. In the Modeling Layer, multiple, different instances of the two metamodels might be created. Any instance of MM1 can be transformed in any instance of MM2, as long as the extends mechanism for the parameters is true. Hence, in case a preceding model is adapted or exchanged, the transformation only needs to be adapted when the inherited attributes change.

The layered approach with the different artifacts allows for reducing complexity and shifting the goal of the models in defined steps through an adaptation of the modeled system architectures incrementally. While the MM1 reflects more the architecture of a ROS 2 system, the MM2 reflects closer the architecture of the proposed formal model. Each parameter reduction and architectural model change is conducted incrementally, reducing the need for complex domain knowledge while allowing traceability. This reflection is essential as an informal proof of modularization to tackle RQ2.

### 3.3 Application of the methodology

To apply the methodology, a toolchain is needed that implements the required components, such as metamodels and transformations. Once the necessary metamodels and transformations are established, the end user (robotics developer) can perform the verification by following the outlined approach. The general flow of applying the methodology is shown in Figure 3, and the workflow can start from two points: verifying legacy systems (Start A) or conceptual systems (Start B).

#### 3.3.1 Verification of legacy systems

Starting with a ROS 2 system implementation (Start A), tracing is used to generate runtime execution traces. These traces are transformed into the ROS 2 Data Model using analysis tools. A parser then converts the ROS 2 Data Model into a model. Multiple model-to-model transformations can be applied within the EMF environment to generate a formal model based on predefined templates. The generated formal model definition is used for formal verification, and the results can guide parameter adjustments in the model or the real system. This iterative process allows for thorough testing before implementing changes in the actual system.

#### 3.3.2 Verification of new systems

For new system designs (Start B), the system can be modeled directly using the created metamodels. This approach allows for system definition without existing source code. By modeling a system using a modeling environment, only the necessary parameters for verification need to be determined, and the code generation can automatically produce the formal model declaration. Iterative verification of system parameters can then be conducted using the models and formal modeling tool, ensuring a robust design before implementation.

### 3.4 Toolchain setup, development, and extension

As described in the previous section, a toolchain is needed to enable the application of the methodology following Figure 3 to verify ROS 2-based applications. The design and implementation of such toolchain is done in a different order than the actual verification process shown in Figure 3. The modularity of components in the methodology allows domain experts to focus on their area of expertise when implementing the toolchain. Each domain expert is responsible for the implementation of specific components and only interacts with other domain experts to implement the connectors, such as transformations. Below, we give an overview of the implementation process of a toolchain that involves several steps, each requiring specific expertise:

Step 1: Development of Formal Models (Formal Methods Expert): In a first step, the formal model component templates need to be defined, created, and tested in the formal modeling tool.
Step 2: Determination and setting of Parameters (Formal Methods Expert/Robotics Expert): Next, the needed input parameters for the formal verification, and how they can be obtained (e.g., through ROS2_tracing), need to be identified. If they cannot be obtained through the mainline analysis tools, additional analysis methods may be required.
Step 3: Development or Adaptation of Tracing Tools (Robotics Expert): In a following step, the tracing needs to be adapted to capture the parameters as required.
Step 4: Development or Adaptation of Metamodels (Software Engineering/Modeling Expert): The next step incorporates an update of the ROS 2 data model and creation of the metamodels needed to cover the components needed for formal verification.
Step 5: Development or Adaptation of Transformations (Software Engineering/Modeling Expert): Implementation of the parsing, the model-to-model, and model-to-text transformations. Create verification models in the formal verification environment and corresponding metamodels.

When creating a toolchain, we assume the goal is the verification using one specific formal model representation. Nevertheless, in case different formal model approaches are to be used, the toolchain can be extended by extending the second and third layer. While the first metamodel in the layer is for a seamless parsing of a trace output to a model in the model environment, the second metamodel enables the transformation towards the formal model representation. Hence, when adding a formal model representation, a further implementation of the second metamodel has to be introduced to adapt to the new formal model. The first metamodel and the parsing can be reused. The possibility of domain experts focusing on defined steps with defined connectors with inputs and outputs in between different layers, is evidence of modularization and helps answering RQ2.

## 4 Toolchain implementation and application

In this section, we apply and evaluate the proposed methodology through the design, implementation, and application of a toolchain. In the first step, we design and implement a toolchain following the process explained in Section 3.4.

### 4.1 Toolchain design

Following the four layers of the proposed methodology, the designed toolchain comprises four main architectural components: three tools (ROS2_tracing, Eclipse/EMF, and UPPAAL) and the actual system implementation.

**FIGURE 5:**
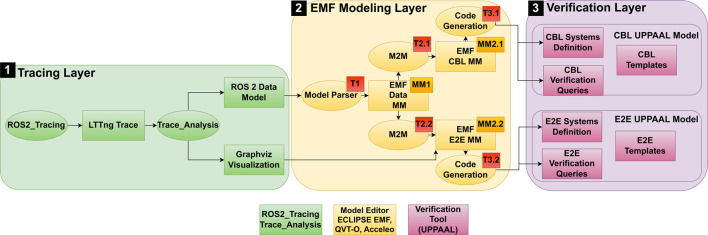
Overview of the proposed toolchain and its components. Note that Layer 0 (ROS 2 Layer) is omitted for space reasons.

An overview of the Tracing Layer, the EMF Modeling Layer and the Verification Layer of the toolchain created in this evaluation is given in Figure 5.

To show the extensibility of the approach, in this paper we implement formal verification based on two approaches of formal modeling. The first approach is verifying callback latency (CBL). The second approach focuses on verification of end to end delays (E2E). Hence, the verification layer comprises of two different UPPAAL models. Each UPPAAL model consists of the UPPAAL timed automata templates, the UPPAAL systems definition and the verification queries.

The parsing layer consists of the tool ROS2_tracing and generated outputs created by the python libraries of tracetools_analysis, such as custom graph visualization.

The modeling layer contains the EMF Data MM, which allows direct parsing of the ROS 2 data model to a model in Eclipse. The model can be reused for the generation of both formal models. Next, the specialized model representation has to be designed for each of the verification approaches individually. Hence, the EMF Modeling Layer contains one metamodel for the CBL and one metamodel for the E2E. Besides the metamodels, the EMF modeling layer contains model transformations.

We implement the transformations,T1,T2.1 and T31 to an extent to be conducted automatically using the chosen tools. Transformations T2.2and T2.3are conducted by hand. Nevertheless, if a feature is contained in the preceding model, it can be contained in the next model after the transformation. Furthermore, some features that are not directly contained can be calculated during the transformation from the parameters that are contained.

In the following, we explain the implementation of the toolchain and the order in which it is designed. Furthermore, we explain the parameters and features that are contained in each element in Figure 5.

### 4.2 Toolchain implementation

In the next sections, we follow the workflow for creation of a toolchain following the methodology presented in Section 3.4.

#### 4.2.1 Step 1: Formal models in UPPAAL

To demonstrate the methodology and toolchain we utilize two different kinds of UPPAAL models for verification. The first kind of UPPAAL models has been created previously (Dust et al., 2023a), and are explained in Section 2. The model focuses on the verification of latency and callback size of a single callback in a ROS 2 system.

**FIGURE 6:**
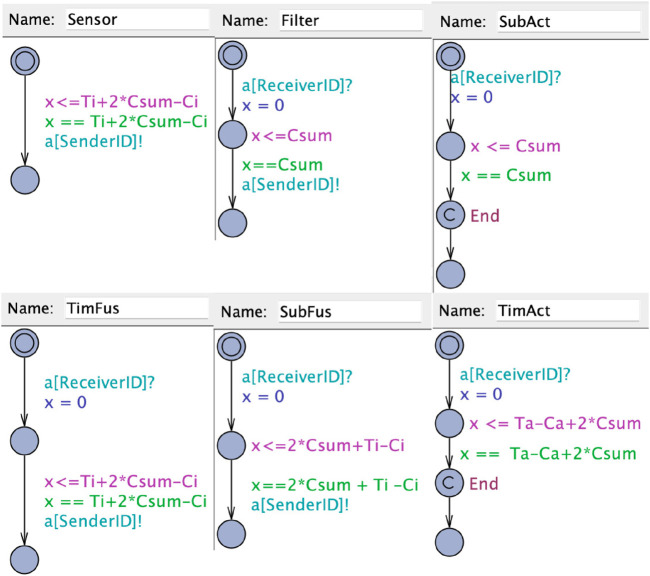
UPPAAL templates for the callbacks.

The second kind of UPPAAL models (E2E) used for evaluation in this paper are created during the implementation of the toolchain. The models aim to allow verification of end-to-end (E2E) latency as proposed in the literature (Teper et al., 2022). Generally, our modeling approach is to create a chain that contains all the components of the original chain and adds delays during the execution that are equally long as the maximum latency for each component. Hence, when executing the model, at the end of the simulation of the last component, the system time will be according to the maximum latency of the chain. In related work (Teper et al., 2022), the authors define six types that can describe a callback based on the function in a processing chain: Sensor, Filter, Timer Fusion, Subscription Fusion, Timer Actuator, and Subscription Actuator. For each of the types of callbacks, we propose UPPAAL templates that can be used to model the end-to-end delays and are shown in Figure 6. In what follows, we give an overview of the proposed templates, where the details, such as included parameters, are explained in Section 4.2.2.

**Sensor**: A sensor node is the start of a chain and sends a message periodically. According to (Teper et al., 2022) the maximum latency is defined as the period minus the maximum execution time plus two times the execution time of all callbacks in the executor. To model the callback in UPPAAL, the callback starts in a location where it waits for the time to elapse until the maximum latency to finish its execution. When the execution of the sensor callback has ended, a following callback is triggered. This is modeled through a synchronized channel to the following callback that is initiated by the sensor.

**Filter**: The subscription actuator can be modeled like a filter, as the maximum latency is equal to two times the sum of all callback execution times (Teper et al., 2022). The callback is modeled by triggering the execution through the synchronized channel through the preceding callback. Upon triggering of the execution, the callbacks stay in the execution location until the maximum blocking time has elapsed. Upon finishing the execution, the next callback is triggered through a synchronized channel.

**Timer fusion**: Timer fusion in a chain basically consists of two relevant callbacks. The first callback receiving a message from the preceding node can be modeled as a filter. The second callback is a timer callback that has the same latency as a sensor (Teper et al., 2022). Hence, we model the callback as a sensor, but with the difference that the callback is triggered by another callback and upon execution triggers another following callback.

**Subscription Fusion**: When modeling a subscription fusion, there are two different paths possible for a chain. If the subscription that triggers the following node of a fusion lies within the same chain (is triggered by a callback in the chain that is modeled). In this case, the callback can be modeled as a filter node.

If the callback that outputs the fusion result is not contained in the same chain, a second chain has to be introduced to determine the maximum latency. As each chain starts with a sensor node, we model the start of that chain as a sensor that is triggered by another node. Following, each element of the same chain including the fusion callback has to be included separately as a fusion callback.

**Timer actuator**: The timer actuator actually consists of two callbacks that have to be modeled accordingly in our UPPAAL representation. The first callback is triggered by the preceding node in the system and resembles a subscription node. In our example, the callback has to be modeled as a filter. The final callback is a timer callback that executes the actuator. This callback has the same latency as a sensor callback (Teper et al., 2022). Hence, we model the callback as a sensor, but with the difference that the execution is triggered by a preceding callback. Upon finishing the execution, the callback passes through an End location that is used for the verification query.

**Subscription actuator**: The subscription actuator can be modeled like a filter, as the maximum latency is equal to two times the sum of all callback execution times (Teper et al., 2022). The only difference is the actuator being the end of the chain. Hence it does not need to trigger another execution of a following callback.

The shown templates are sufficient to model processing chains of callbacks and determine the upper bound for the latency by checking the global system time while the Actuator callback passes through the End location.

#### 4.2.2 Step 2: Determining parameters, features, and formal model declaration

In this step, we identify the parameters required to instantiate the UPPAAL templates for model verification. These parameters are essential for accurately modeling the system’s behavior and ensuring the correctness of the verification process.

##### 4.2.2.1 Buffer overflow UPPAAL templates

The legacy UPPAAL model includes three types of templates: BufferOverflow, Executor, and Callbacks. Each template requires specific parameters to be instantiated, as follows.

###### 4.2.2.1.1 Executor template

• **stoptime**: The time at which the executor stops executing. This parameter defines the duration for which the verification is conducted.

###### 4.2.2.1.2 PeriodicCallback template

• **id**: A unique identifier for the callback.

• **execution time (Ci)**: The time required to execute the callback.

• **period (Ti)**: The interval at which the callback is triggered.

• **type**: The type of callback (e.g., timer, subscriber).

• **buffer size**: The size of the buffer associated with the callback.

###### 4.2.2.1.3 SporadicCallback template

• **id**: A unique identifier for the callback.

• **execution time (Ci)**: The time required to execute the callback.

• **amount of releases**: The number of times the callback is released.

• **release array**: An array specifying the release times of the callback.

• **type**: The type of callback (e.g., timer, subscriber).

• **buffer size**: The size of the buffer associated with the callback.

##### 4.2.2.2 End-to-end (E2E) timing analysis templates

For end-to-end timing analysis, we use several templates to model different components of the system. Each template requires specific parameters to capture the timing behavior accurately.

###### 4.2.2.2.1 Sensor template

• **Csum**: The sum of the execution times of all callbacks in the system/executor.

• **Ci**: The execution time of the callback.

• **Ti**: The period of the callback.

• **SenderID**: A unique identifier for the callback that sends information.

###### 4.2.2.2.2 Filter template

• **Csum**: The sum of the execution times of all callbacks in the system/executor.

• **ReceiverID**: The identifier of the callback that receives information.

• **SenderID**: The identifier of the callback that sends information.

###### 4.2.2.2.3 SubFus template

• **Csum**: The sum of the execution times of all callbacks in the system/executor.

• **Ci**: The execution time of the callback.

• **Ti**: The period of the callback.

• **ReceiverID**: The identifier of the callback that receives information.

• **SenderID**: The identifier of the callback that sends information.

###### 4.2.2.2.4 TimFus template

• **Csum**: The sum of the execution times of all callbacks in the system/executor.

• **Ci**: The execution time of the callback.

• **Ti**: The period of the callback.

• **ReceiverID**: The identifier of the callback that receives information.

• **SenderID**: The identifier of the callback that sends information.

###### 4.2.2.2.5 SubAct template

• **Csum**: The sum of the execution times of all callbacks in the system/executor.

• **ReceiverID**: The identifier of the callback that receives information.

###### 4.2.2.2.6 TimAct template

• **Csum**: The sum of the execution times of all callbacks in the system/executor.

• **Ci**: The execution time of the callback.

• **Ti**: The period of the callback.

• **ReceiverID**: The identifier of the callback that receives information.

###### 4.2.2.2.7 Explanation of parameters

• **Ci (execution time)**: The time required to execute a callback.

• **Ti (period)**: The interval at which a callback is triggered.

• **SenderID**: A unique identifier for the callback that sends information, ensuring it can be correctly identified.

• **ReceiverID**: Matches the receiving callback with the sender, ensuring proper communication between callbacks.

• **Csum**: The cumulative execution time of all callbacks within the system or executor, used to assess overall system load.

After determining the described parameters, we can instantiate the UPPAAL templates to create a model that allows for formal verification of buffer overflows and end-to-end latency, via model checking.

#### 4.2.3 Step 3: Setup of ROS2_tracing and graph analysis

In a trace generated by ROS2_tracing, the execution of each individual callback is contained. Hence, the callbacks and their executions can be mapped from the tracing output to the model. The timing information contained in the traces allows the calculation of the maximum execution time for each callback. Additionally, for timers, the configured period is recorded in the traces. By aggregating the execution times of all callbacks, the total execution time for the system can be calculated, with the assumption that all callbacks are executed within the same executor.

To model the callback chain, we utilize the tracing information to identify publishers and subscribers, along with their preceding and following nodes. The primary challenge lies in determining the type of each callback for accurate modeling. In the initial automated transition, we categorize all receiving and sending callbacks as filters, all timers as sensors, and all sinks as actuators. A message flow analysis is conducted to visualize the internal connections and relationships, which helps in manually constructing the graphs.

#### 4.2.4 Step 4: Development of metamodels

To follow the process shown in Figure 5, we implement three different Metamodels that can be used to create models containing the information needed to automate the verification. MM1 is used to parse the tracing output with all its information into a model of the same architecture to allow traceability.

MM2 is the EMF CBL Metamodel allowing to create models that resemble the system architecture for individual node verification and MM3 is the EMF E2E metamodel allowing modeling of a system to transform it into UPPAAL code for verification of the E2E latency. In the following, we show the implementation of the metamodels.

##### 4.2.4.1 Metamodel 1 - Eclipse Data Metamodel

**FIGURE 7:**
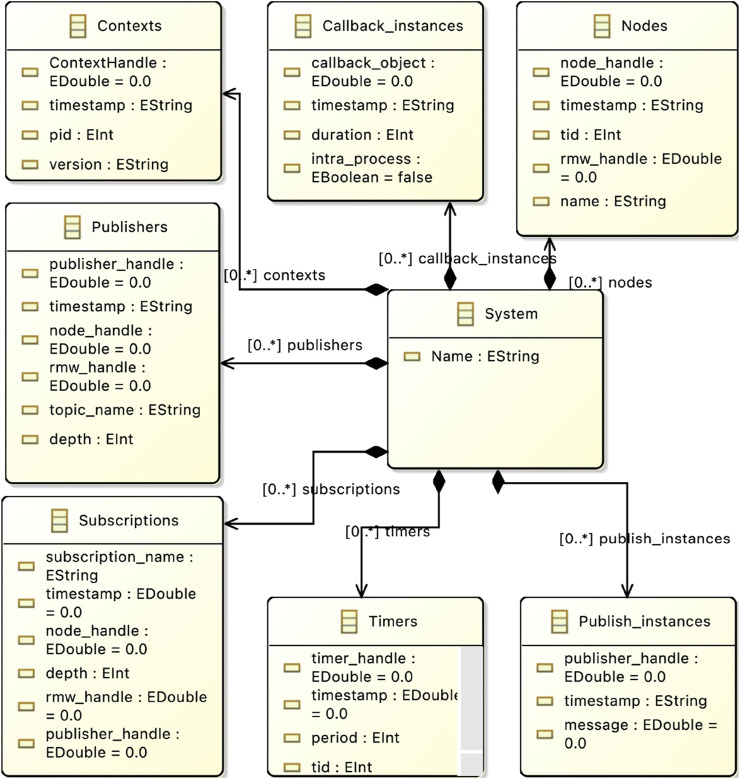
Data Metamodel in Eclipse.

In the initial step, we develop the EMF Data metamodel, which includes all system components specified by the ROS 2 Data Model, such as subscriptions, callbacks, and timers, represented as classes with parameters as attributes. Figure 7 shows an excerpt from the metamodel implementation. The yellow boxes indicate the classes in the metamodel, with arrows illustrating the dependencies between them. All classes representing system components are child objects of a master class that represents the entire system. Although in a ROS 2 implementation, components like Publishers are contained within Nodes, in this metamodel, the association of Publishers to Nodes is managed by identification handlers modeled as attributes. This approach aligns the representation of the ROS 2 Data Model with the EMF data model.

##### 4.2.4.2 Metamodel 2 - EMF CBL Metamodel

**FIGURE 8:**
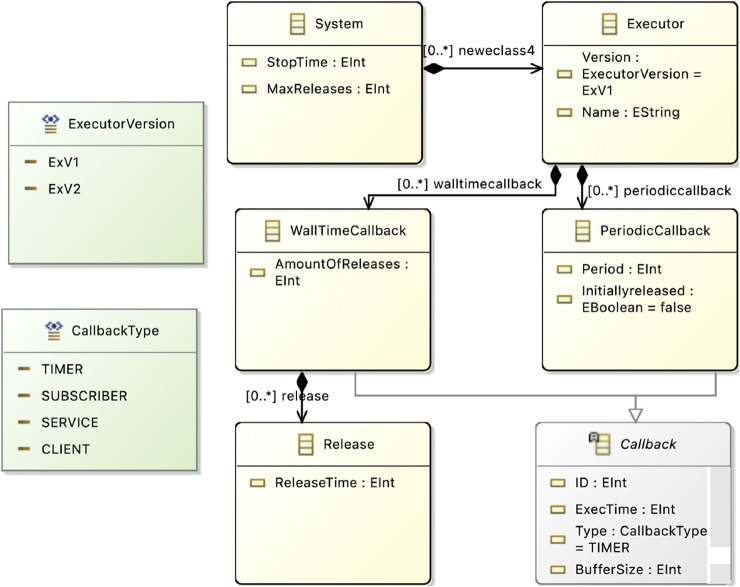
Verification Metamodel in Eclipse.

The second metamodel represents the UPPAAL templates within the EMF framework, which are utilized for formal verification. An overview is provided in Figure 8.

In this metamodel, each of the three UPPAAL templates (Executor, WallTimeCallback, and PeriodicCallback) is represented as a distinct class, which are components of a system. As detailed in Section 2, each template includes parameters such as *ids* and buffer sizes. Additionally, the metamodel defines datatypes for attributes like callback type and executor version, which can be specified in a model instance.

Although the toolchain proposal mentions the modeling of requirements such as maximum callback latency, this aspect is reserved for future work.

##### 4.2.4.3 Metamodel 3 - EMF E2E Metamodel

**FIGURE 9:**
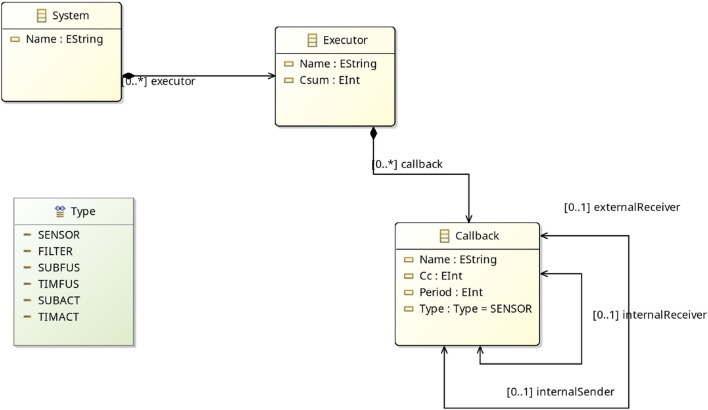
E2E Metamodel in Eclipse.

In the created metamodel in Figure 9, the callbacks are modeled as a single class contained in an executor that is part of a system. The callbacks are distinguished by their Type, which is a parameter. Furthermore, the parameters needed to initialize the UPPAAL model such as execution times and the sum of callback execution times. This model allows the representation of the features needed for the end-to-end verification.

#### 4.2.5 Step 5: Implementation of model-to-model transformations

##### 4.2.5.1 T1: Model parsing - ROS 2 to EMF Data Metamodel

We implement the parsing T1 as a python function in Trace _ Analysis. The function takes the ROS 2 Data Model and creates an XML file that can be imported as a model (using the EMF Data Metamodel) in the Eclipse workspace. The parsing is done by reading the data of the ROS 2 Data Model and printing them in an XML document with the desired formatting of the EMF Data Model.

##### 4.2.5.2 T2.1: Model-to-Model - EMF Data Metamodel to EMF CBL Metamodel

In this model-to-model transformation, T2.1 the components of the EMF Data Model are mapped to the components in the verification model using QVT-O. QVT-O is an operational mapping language (Eclipse Foundation, 2025b). In the first step, the periodic timers are mapped to periodic callbacks with the type attribute set to TIMER. The period and execution time is extracted from the attributes in the EMF Data Model and passed to the verification model. Furthermore, for timers, the buffer size is set to one. The second mapping is between the subscription callback and the wall-time-callback of the type SUBSCRIBER. A subscription callback is released on the reception of data. As neither the release time of the callback, nor the reception of the data is initially contained in the system traces, we map the publishing time of the data in the topic the callback is subscribed to as the release time. Furthermore, we pass the execution time and buffer size as further parameters.

##### 4.2.5.3 T3.1: Model-to-Text - EMF CBL Metamodel to UPPAAL code

With the model-to-text transformation T3.1 using the tool Acceleo (Eclipse Foundation, 2025a), the EMF verification model is translated into the UPPAAL code. Therefore, the classes contained in the EMF verification model are mapped to specific code snippets, e.g., representing the UPPAAL template instantiation. Then, the code is dynamically filled with the needed parameters based on the class attributes.

##### 4.2.5.4 T2.2, T3.2: EMF Data Metamodel to EMF E2E Metamodel to E2E UPPAAL code

The transformations T2.2 and T3.2 are needed to fully automate the process of verifying ROS 2 applications using the E2E UPPAAL models. As we evaluate the automation of such transformations on T2.1 and T3.1, and the repetitive implementation of the transformation as being rather engineering than research, for the sake of simplicity, T2.2 and T3.2 are not implemented by a tool but, in the context of this paper, are conducted manually.

### 4.3 Toolchain application

The application and evaluation of the toolchain is carried out using three ROS 2 systems (Use-Case 1, 2, 3). Use-Case 1 and Use-Case 3 are implemented in source code and executable, while Use-Case 2 is evaluated from a conceptual perspective without actual source code implementation.

We show the automation of formal verification on two examples of formal models in UPPAAL, each focusing on a different set of properties to verify. The implementation shows how to set up the toolchain and its main components in the context of verification of *buffer overflow*, *callback latency* and *end-to-end delays* for ROS 2 processing chains.

While for the verification of the buffer overflow and callback latency, we reuse UPPAAL templates that have been proposed in related work, for the verification of end-to-end timing analysis, we propose new UPPAAL UTA templates. We implement exemplar metamodels to demonstrate the application of the toolchain. For the verification of the buffer overflow, we implement prototypes of the model parsing, the model-to-model transformation, and the model-to-text transformation. In the second part of verifying end-to-end latencies, we perform the transformations by hand. The created artifacts, such as metamodels, templates, source code, and graphs, are published in (Dust et al., 2025).

#### 4.3.1 Use-cases

The first system (Use-Case 1) is a lightweight ROS two implementation of two nodes similar to the setup depicted in Figure 2. The system has been proposed by Casini et al. (2019) and used for real-time evaluation and demonstration of different scheduling approaches of ROS two in Blaß et al. (2021), Dust et al. (2023b), and Dust et al. (2023a). The system offers traceability through controlled execution times and controlled trigger events of the included callbacks. The limited complexity simplifies manual analysis, and hence enables simpler comparison and evaluation of verification and modeling approaches.

**FIGURE 10:**
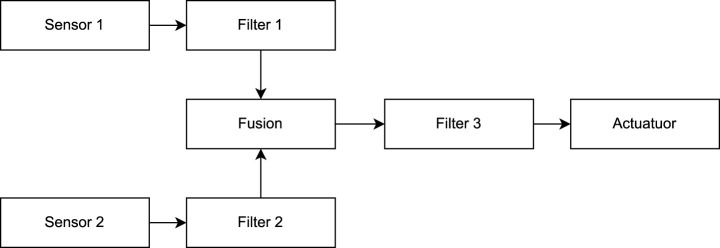
System overview.

**FIGURE 11:**
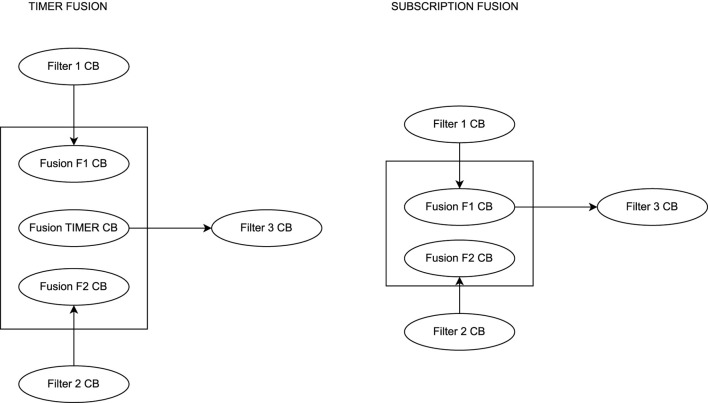
Two versions of the fusion node.

**FIGURE 12:**
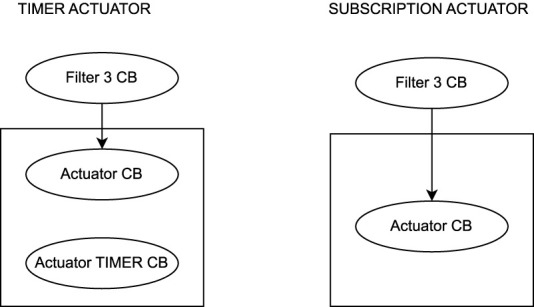
Two versions of the actuator node.

The second system (Use-Case 2) is a conceptual ROS two system as given in the evaluation of Teper et al. (2022). The system is used to evaluate the correctness of the created UPPAAL templates for E2E verification. As a part of RQ3 we aim to provide validated UPPAAL models. The correctness of the proposed formal models is demonstrated by repeating the calculations from the case study in Teper et al. (2022). Furthermore, as the system is a conceptual design, as we have no access to the original ROS two code, the approach of verification of conceptual designs (START B) is demonstrated. An overview of the nodes in the system is given in Figure 10. The system consists of two sensors, which contain a ROS two timer each publishing a message at a given interval. The message is received by a filter callback that forwards the message on reception. The two filter messages are fused into one message in the fusion node. A third filter node receives and forwards the fused message. The final message is received by an actuator node. In an actual system, the fusion and the actuator can be implemented in two different ways. The subscription fusion and actuation, and the timer subscription and actuation. Both are shown in Figures 11, 12. In the subscription configuration, the messages are forwarded using the same callback triggered by the subscriptions. In the timer configuration, the messages are received by a subscription callback, and then the final message is published by a different timer callback.

**FIGURE 13:**
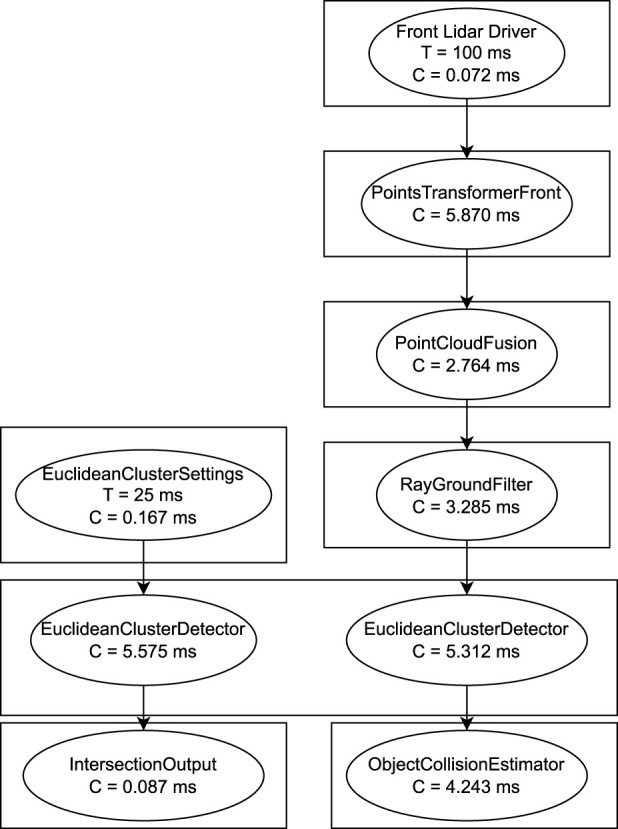
Extract from the lidar pipeline in the autoware reference system with the obtained worst case execution times C and the periods T for timers.

The third system (Use-Case 3) is an ROS two real-time benchmark system (ROS Realtime Working Group, 2025b) and resembles parts of an autonomous driving stack. As a controlled real-world system is used to demonstrate applicability of the proposed methodology. In Figure 13, we show an excerpt of the system, visualized through implemented message flow analysis. For simplicity, we focus the verification of the end-to-end latency on the chains shown in the figure.

#### 4.3.2 Application of legacy UPPAAL models and automated transformations on use-case 1

As a first step, we utilize Use-Case 1 to assess and validate our toolchain prototype regarding automation as stated in RQ1, and verification as stated in RQ3. We evaluate the proof of concept by generating UPPAAL code through our proposed workflow, with an example excerpt shown in Listing 1. Instead of manually analyzing the system and calculating potential release times, our approach automatically generates traces and performs model transformations to produce executable UPPAAL code. We then ran this code in UPPAAL and verified the accuracy of the results at each stage.

By focusing on subscription and timer callbacks, the generated traces provided the necessary information to either directly determine or infer the required parameters, such as callback periods, release times, buffer sizes, and execution times. However, some parameters for services and clients, as well as certain subscription callback release times, are currently not captured by ROS2_tracing.

Despite these limitations, the presence of parameters for subscribers and periodic timers demonstrates the feasibility of our toolchain. Additionally, it is possible to add custom trace points, although this requires expert knowledge. We are working on incorporating the necessary trace points into the mainline releases of ROS 2.

Our observations indicate that the model-based verification of ROS two applications can be automated by using system execution traces and model-driven engineering to automatically populate model-based verification templates.

#### 4.3.3 Verification of E2E latency of conceptual system design on use-case 2

**FIGURE 14:**
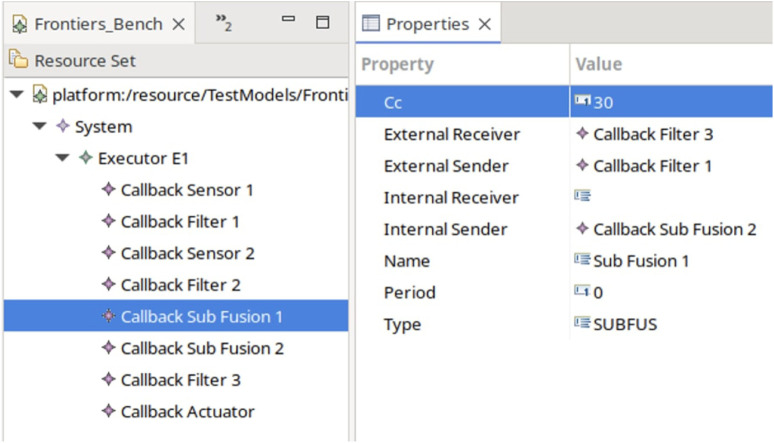
E2E Model instance in Eclipse.

To implement the system in the modeling layer, we create models in Eclipse containing the different configurations following the grammar of the defined E2E metamodel. An example of such a model for a subscription fusion and the subscription actuation is shown in Figure 14. It can be seen that all callbacks are included in the same executor. Furthermore, the callbacks contain information such as the period and type of timers. The callback, whose parameters are shown in the example, is the Fusion F1 CB that receives the data from Filter one and forwards the fused message to Filter 3. The links are done through the connection of the parameters External Receiver and External Sender. The fusion is indicated through the internal sender Sub Fusion 2.

Next, we transform the model into the system instantiation for UPPAAL as shown in Listing 2 for an example with a subscription fusion and subscription actuation. As described, the actual transformation in this example is done by hand, but can be automated through Acceleo model-to-text transformation.

Listing 2 Created UPPAAL model system declaration.

// Under Utilized System: Sub Fus, Sub Act
// Csum: 180ms, Cch1: 110ms, Cch2 160ms, Ts 360ms
// Ct1: 10ms Tt1: 360ms
// Ct2: 20ms Ct2: 360ms
// CHAIN 1:
/*
S1 = Sensor(180, 10, 360, 0);
F1 = Filter(180, 0, 1);
Fus1 = Filter(180, 1, 2);
F3 = Filter(180, 2, 3);
A = SubAct(180, 3);
system S1, F1, Fus1, F3, A;
*/
// CHAIN 2:
/*
S2 = Sensor(180, 20, 360, 0);
F2 = Filter(180, 0, 1);
Fus2Cb = Filter(180, 1, 2);
S1 = SubFus(180, 10, 360, 2, 3);
F1 = Filter(180, 3, 4);
Fus1 = Filter(180, 4, 5);
F3 = Filter(180, 5, 6);
A = SubAct(180, 6);
system S2, F2, S1, Fus2Cb, F1, Fus1, F3, A;
*/

After performing the transformations and modeling in UPPAAL, we run the verification and compare the obtained results for the E2E latency with the results from (Teper et al., 2022). Table 1 shows the system parameters and the results of the verification, which match the results from Teper et al. (2022). Hence, we validate the correctness of our models to achieve parts of our goal in RQ3 and the approach to the transformations towards RQ1 with the verification of conceptual system designs.

**TABLE 1:** Experiment Results with the formal models (all results are in ms).

		Csum	Cch	Ts	Tf	Ta	Teper et al.	Formal models
sub fus + sub act	Chain 1	180	110	360	—	—	1,430	1,430
	Chain 2	180	160	360	—	—	2,490	2,490
sub fus + tim act	Chain 1	210	140	420	—	840	2,900	2,900
	Chain 2	210	190	420	—	840	4,140	4,140
tim fus + sub act	Chain 1	210	140	420	840	—	2,900	2,900
	Chain 2	210	160	420	840	—	2,890	2,890
tim fus + tim act	Chain 1	240	170	480	960	960	4,730	4,730
	Chain 2	240	190	480	960	960	4,720	4,720

#### 4.3.4 Verification of E2E latency of legacy system implementation on use-case 3

In the following, we use the implementation of the ROS 2 Autoware Real-Time benchmark (ROS Realtime Working Group, 2025b) (Use-Case 3) to show the verification process on an actual ROS two implementation. In this experiment, we demonstrate formal verification on a real-world use-case to answer RQ3. Furthermore, we demonstrate the modularization of the toolchain and reusability of components compared to the application of Use-Case 1 to answer RQ2. We run the system in a development container and create a ROS2_tracing trace. The trace is transformed into the ROS 2 Data Model. In Figure 13, we show an excerpt of the system, visualized through implemented message flow analysis. For simplicity, we focus the evaluation on the chains shown in the figure. Furthermore, we assume all the callbacks to be in the same executor.

We perform verification using the E2E metamodels. We transform the system into four different versions of the verification metamodel. Each representing a different chain that can be verified from the given example. The first chain is going from the Euclidean Cluster Settings to the Intersection Output. The second chain goes from the Front Lidar Driver to the Object Collision Estimator. The third chain goes from the Front Lidar Driver to the Intersection Output via subscription fusion on Euclidean Cluster Detector. The last chain goes from the Euclidean Cluster Settings to the Object Collision Estimator via subscription fusion on the Euclidean Cluster Detector. Hence, following the methodology from Start A, the following results in Table 2 are obtained for the E2E latency for the individual chains.

**TABLE 2:** Obtained Upper bounds for the End-To-End Latency.

Chain	E2E Latency
C1	134.333 m
C2	291.553 m
C3	398.511 m
C4	398.511 m

## 5 Evaluation and discussion

In this section, we first compare the proposed methodology and implemented toolchain with a manual application of formal verification. Next, we discuss the threats to validity before answering the research questions.

### 5.1 Comparison of manual and automated verification

After implementation and application of the toolchain, in this section, we compare the verification steps following the methodology to a manual approach to provide more evidence towards automation in RQ1 and modularization for RQ2.

#### 5.1.1 Verification of legacy systems

The following steps are needed to perform formal verification of legacy systems using a manual approach:

1. System Implementation
2. System Component Determination
3. Real-Time Parameter Determination
4. Formal model System composition
5. Formal Verification

The following steps are needed to perform formal verification of legacy systems using the automated approach:

1. System Implementation
2. Systems Execution and Tracing
3. Model Parsing
4. Model Transformation
5. Verification Code Generation
6. Formal Verification

At first glance, the automated verification approach contains more steps. Nevertheless, the given steps can be performed using pre-defined transformations. In the manual approach, analysis and extraction of system components and runtime parameters have to be conducted by hand, which is error-prone and requires application and domain knowledge. Furthermore, additional domain expert knowledge in formal verification is needed to apply formal modeling and verification.

#### 5.1.2 Verification of conceptual systems

The following steps are needed to perform formal verification of conceptual systems using a manual approach:

1. Formal Model System Definition
2. Real-Time Parameter Determination
3. Formal Verification

The following steps are needed to perform formal verification of conceptual systems using the automated approach:

1. Architectural Modeling
2. Real-Time Parameter Determination
3. Generation of Formal Models
4. Formal Verification

In a manual approach to verifying conceptual systems, the practitioner directly works in the formal modeling environment. This reduces the complexity of the toolchain. However, domain knowledge is required to create formal models and formal verification. In the automated approach, a practitioner models the system in a modeling environment before automatically generating the formal models using pre-defined transformations.

### 5.2 Limitations and threats to validity

While the proposed methodology has strong potential for generalization due to its use of MDE techniques, the need for customization and the reliance on specific tools during the implementation present challenges.

First of all, there might be scenarios where transformations from ROS two execution traces to formal models (via EMF metamodels) may not fully capture all relevant system behaviors and parameters. Additionally, ROS2_tracing may not capture all necessary parameters (e.g., service/client interactions), which could lead to incomplete or incorrect models.

Next, the automated classification of callbacks and system components (e.g., as filters, sensors, actuators) based on trace data may lead to misclassifications, especially in complex chains.

In the implementation of the methodology, there is a toolchain dependency, where the correctness of the verification heavily depends on the accurate functioning of multiple tools (ROS2_tracing, Eclipse EMF, QVT-O, Acceleo, UPPAAL). Bugs or misconfigurations in any of these could compromise results.

In this paper, a proof of concept is demonstrated on a conceptual system and a specific benchmark (Autoware). The scaling of the approach to large, heterogeneous, or multi-executor ROS two systems has to be evaluated further. As an example, the evaluation in this paper assumes all callbacks are in the same executor, which may not reflect real-world deployments with multiple executors or distributed systems.

Additionally, the toolchain and formal models rely on specific versions or configurations of ROS two and the tracing tools used, limiting applicability across different setups. Nevertheless, different templates of formal models can be introduced to model different versions of ROS two systems that can be matched through manually set parameters by the developer.

While the toolchain is shown to work in a controlled setting, there is a need for extended statistical or empirical analysis to support claims of improved efficiency, accuracy, or usability. Furthermore, user studies or usability evaluations are needed to evaluate the simplifications for robotics developers when applying formal methods. Furthermore, more evaluation is needed on how verification results are interpreted or used to guide system design decisions.

### 5.3 Answers to the research questions

After utilizing the methodology to implement and apply formal verification, in this section, we answer the posed research goals.

**RQ1:** What approach can be employed to automate the application of formal verification of ROS 2-based applications?

Answering this question, we first propose a methodology that incorporates four layers. The first layer is the implementation of ROS two application. In case an implementation is given, the system can be run, and runtime information can be recorded during execution in an execution trace. Such a generated trace can be utilized to parse the given system and run-time parameters into a model in a chosen modeling environment. In the modeling environment, a second metamodel allows the focus on needed parameters and components for formal verification. To allow automation in the modeling process, model-to-model transformations can be utilized to automatically create a model instance of the second model based on an instance of the first model. The second model can then be automatically transformed to the formal model representation utilizing formal model templates and a model-to-text transformation.

Generally, while the traces could be parsed into the second model representation or even the formal model directly, the introduction of the second layer of modeling allows extendability and decouples the process of tracing from the modeling and verification.

Following the methodology, verification can not only be automated with a given ROS two system implementation, but the model-to-text transformation can be used to generate a formal model from the EMF model automatically. This allows users to start with conceptual systems design in the modeling environment before implementing a system.

To demonstrate and assess the automation, we implement a toolchain with the model parsing, the model-to-model, and a model-to-text transformation for verification of callback latency.

We use the tool ROS2_tracing to generate system traces, which are converted into a ROS two data model using trace_analysis. We extend trace_analysis by a function that allows automated parsing of the data model to an EMF instance of the same data model in Eclipse.

We implement model-to-model transformation utilizing QVT-O and test the generation of the second EMF model representation. Next, we implement and test the model-to-text transformation using Acceleo, where we generate runnable UPPAAL code that is used for formal verification.

Hence, we show that utilizing model-driven engineering techniques with model parsing, model-to-model, and model-to-text transformation can automate the process of determining the parameters and, secondly, the instantiation of formal models.

Furthermore, in the same toolchain, we implement models to verify end-to-end latency in ROS two processing chains. In this implementation, the model parsing and the EMF data model can be reused. We implement the second model and the UPPAAL templates. The model-to-model and model-to-text transformations have not been implemented and have only been conducted by hand. Nevertheless, we demonstrate the automation of such transformations on the first verification example. Hence, in future work, the transformations can be automated as well.

**RQ2:** How can the formal verification process be modularized to enable domain experts to concentrate on their specific areas of expertise without requiring deep formal methods knowledge?

In this methodology, we apply model-driven engineering to decouple the process of formal modeling from systems tracing and automate significant steps throughout the process. We implement two different metamodels for each formal verification approach. The first metamodel allows the import of parameters obtained by tracing and does not need to be adapted until the tracing approach changes. The second metamodel focuses on the parameters needed in a specific verification approach. Hence, such a metamodel is added or changed when formal models are adapted or added. Two consecutive models are connected by the fact that the first model is an extension of the second model. The models are substitutable by any other model that obeys the extension mechanism. However, the transformation needs to be adapted, provided that the attributes of the substituted class change. This acts as an informal proof of modularization. In the implementation of the toolchain, we demonstrate such ability of replacement by implementing verification of ROS two systems using two different UPPAAL models. Each of the UPPAAL models has its own implementation of MM2, but stems from the same implementation of MM1.

Following the proposed methodology, we identify three main expert domains that are needed in the creation and maintenance of a toolchain.

1. Robotics Expert: Following the given methodology, the robotics expert is responsible for the tracing of systems and runtime parameters, such as the application of the final toolchain.
2. Software Engineering/Modeling Expert: The software engineering/modeling expert implements the model-to-model transformations, such as the definition of the metamodels. The implementation of the parsing and the model-to-text transformations needs to be in collaboration with the robotics expert (parsing) and the formal methods expert (model-to-text).
3. Formal Methods Expert: The formal methods expert is responsible for the creation of the formal model templates that can be reused for verification. Furthermore, the expert needs to compose the formal verification queries.

As the transformations between the toolchain components can be automated, the robotics developer as a practitioner only needs to learn the execution of such transformations to apply a toolchain following the proposed methodology.

**RQ3:** How can a methodology incorporate verification using different formal models?

With the last research question, we focus on the modularity and extensibility of the toolchain. The proposed methodology incorporates multiple steps in a modeling environment. Firstly, this allows the decoupling of domain expertise needed to design such a component, but it also allows for the extendability of the toolchain. The model parsing and implementation of the ROS two data model, such as the EMF data model, is reusable for different verification approaches. As long as the parameters are contained in the trace, multiple formal model representations can be built upon such parameters. To introduce a different formal model representation to the toolchain, we add a different EMF model in the second part of the toolchain. This EMF model represents the parameters and components needed for the second formal model representation. To automate the verification process, new model-to-model and model-to-text transformations have to be created, incorporating the new EMF model representation and the UPPAAL templates. When applying the methodology to multiple formal model representations in the same toolchain, such a toolchain consists of one tracing and parsing and data model implementation that can be reused for all formal model representations, as long as all needed parameters are contained in the trace. Next, there will be their formal models and individual EMF metamodels for each of the individual approaches with their specific model-to-model and model-to-text transformations. Hence, when extending the toolchain with a new formal model, the methodology can be applied to extend an existing toolchain with the needed components, while reusing the model parsing and the EMF data model.

## 6 Related work

The analysis of ROS two execution behavior has been a subject of interest in recent research. Casini et al. (2019) and Blaß et al. (2021) conduct response time analysis, which is crucial for the formal verification of ROS two timing behavior. Their work has laid the foundation for creating formal model templates and modeling timing requirements.

Halder et al. (2017) propose formal verification of ROS two communication between nodes using UPPAAL. Their approach models low-level parameters such as queue sizes and timeouts to verify queue overflow. While their focus is on modeling and verification, our toolchain emphasizes the automation of verification processes.

Carvalho et al. (2020) employ an Alloy extension called Electrum to implement a model-checking technique that automatically creates models from configurations extracted in continuous integration and specifications. Their approach targets high-level architectural verification, whereas our toolchain aims to verify low-level behavior such as system execution.

Webster et al. (2016) and Liu et al. (2018) work on formal verification of requirements for robotic systems and ROS two message passing in DDS using different model checkers. Although their approaches are manual and use different model checkers than UPPAAL, our focus is on automating the verification process. Extending our toolchain to support other model checkers could be a valuable future direction.

Kim and Kim (2022) introduce the Robo Fuzz Framework, which is used for fuzz testing robotic systems to find bugs in system implementations. Their framework focuses on data type mutation and violation of physical laws and hardware specifications. In contrast, our framework focuses on timing and execution verification of ROS two applications. Additionally, fuzz testing is not exhaustive.

Anand and Knepper (2015) present ROSCoq, a “correct-by-construction” approach for developing certified ROS two systems. While their approach is not applicable to legacy systems, it complements the verification conducted in our work.

Beckmann and Thoss (2010) explore model-based development of DDS-based systems such as ROS 2. The work highlights how the DDS architecture supports model-based development, whereas our focus is on verification.

Parra et al. (2021) develop Ecore models to specify QoS requirements for ROS 2. This work is complementary to ours, as we focus on architectural components and verification related to task scheduling.

Dal Zilio et al. (2023) propose a toolchain for runtime and offline verification of general robotic systems beyond ROS. While the authors focus on general robotic systems and application code with internal logic, our toolchain targets timing issues induced by using ROS 2 as middleware. Additionally, our toolchain leverages model-driven engineering in the Eclipse environment to support iterative verification and model-based development.

Teper et al. (2022) provide an end-to-end timing analysis for ROS two systems, focusing on cause-effect chains and their timing behavior. The work is significant for understanding the maximum reaction time and maximum data age in ROS two systems, which are critical for ensuring real-time performance.

Bédard et al. (2023) utilize ROS2_tracing to allow message flow analysis of ROS two systems. The work can be used as a ground for allowing additional analysis in the tracing layer of our toolchain, and is used as a foundation for the structural analysis of ROS two systems in our evaluation.

In our previous work (Dust et al., 2023a), we develop reusable UPPAAL templates to verify timing behavior and buffer overflow in ROS two systems. Building on this foundation, our current work aims to further simplify the formal verification process by automating parameter determination and model initialization, making formal verification more accessible and less error-prone for robotics developers.

## 7 Conclusion and future work

In this article, we introduce a novel approach to automating model-based verification for ROS 2-based applications using model-driven engineering techniques. This work extends our paper (Dust et al., 2024) and builds on our previous work (Dust et al., 2023b), which identified potential timing issues, and utilizing the formal model templates proposed in (Dust et al., 2023a). In this article, we develop a methodology that leverages ROS two system traces to automate the verification process. Our toolchain uses ROS two execution traces to initialize pre-defined formal model templates through models and model transformations.

The toolchain supports the verification of both implemented and conceptual systems by providing four different model representations, enhancing traceability throughout the process. Additionally, it allows for parameter refinement and iterative verification of system parameters without repeated source code adaptation.

A key feature of our approach is its flexibility in supporting different types of formal modeling analyses. We demonstrated this by comparing two formal modeling approaches: one at the individual node level and one at the system level (end-to-end analysis). The individual node level analysis focuses on verifying the timing behavior of specific nodes, while the end-to-end analysis examines the timing behavior of cause-effect chains across the entire system. This comparison showcases the toolchain’s versatility in accommodating various verification needs.

Our evaluation demonstrates the feasibility of using ROS2_tracing to capture the necessary trace points for verification. However, customization may be required to include all needed parameters. Further evaluation is needed to determine the extent to which execution times from a single system execution are sufficient for verification. Nonetheless, our work shows the potential for automatic parameter determination using system traces and model-based development for formal verification.

The toolchain also opens up possibilities for automated model-based generation of ROS two application code and modeling of requirements, which are areas for future research. While these features would enhance automation, they are not essential to demonstrate the feasibility and novelty of our methodology.

Despite being demonstrated with specific tools (ROS2_tracing, Eclipse EMF, Acceleo, QVT-O, and UPPAAL), our approach can be implemented using different tools, such as other tracing tools, model editors, and verification tools. This flexibility makes our methodology adaptable to various development environments. As an example, when choosing a different formal modeling environment, only an additional metamodel with the corresponding model-to-model and model-to-text transformations is needed. In contrast, the tracing and parsing components do not need to be changed.

Given the preliminary state of the toolchain implementation, this paper serves as a first proposal and proof of feasibility, making it suitable for researchers and tool developers. More evaluation and implementation are needed to make the toolchain usable on real robotics systems. To enable more extensive verification, the proposed metamodels and transformations need to be refined and extended. Additional formal model templates should be developed and integrated into the toolchain. Future work will also involve modeling requirements and verification properties, which were not included in this implementation.

In conclusion, our work demonstrates the potential of using system traces and model-driven engineering to automate the formal verification of ROS two systems. By refining the data model and output of ROS2_tracing, and providing formal proof of correctness for the toolchain implementation, we can further enhance the robustness and usability of our approach. Future research will focus on automating verification and simulation feedback, modeling requirements, and generating ROS two application code. Additionally, investigating the ease of use of the proposed toolchain will be essential to ensure its practical applicability in real-world scenarios.

## Data Availability

The datasets presented in this study can be found in online repositories. The names of the repository/repositories and accession number(s) can be found below: https://sites.google.com/view/mbfvros2.
